# Measuring kinetic drivers of pneumolysin pore structure

**DOI:** 10.1007/s00249-015-1106-x

**Published:** 2016-02-23

**Authors:** Robert J. C. Gilbert, Andreas F.-P. Sonnen

**Affiliations:** Division of Structural Biology, Wellcome Trust Centre for Human Genetics, University of Oxford, Roosevelt Drive, Oxford, OX3 7BN UK; European Molecular Biology Laboratory, Structural and Computational Biology Unit, Meyerhofstraße 1, 69117 Heidelberg, Germany

**Keywords:** Pore formation, Kinetics, MACPF/CDC, Toroidal pore, Oligomerization, Membrane structure

## Abstract

Most membrane attack complex-perforin/cholesterol-dependent cytolysin (MACPF/CDC) proteins are thought to form pores in target membranes by assembling into pre-pore oligomers before undergoing a pre-pore to pore transition. Assembly during pore formation is into both full rings of subunits and incomplete rings (arcs). The balance between arcs and full rings is determined by a mechanism dependent on protein concentration in which arc pores arise due to kinetic trapping of the pre-pore forms by the depletion of free protein subunits during oligomerization. Here we describe the use of a kinetic assay to study pore formation in red blood cells by the MACPF/CDC pneumolysin from *Streptococcus pneumoniae*. We show that cell lysis displays two kinds of dependence on protein concentration. At lower concentrations, it is dependent on the pre-pore to pore transition of arc oligomers, which we show to be a cooperative process. At higher concentrations, it is dependent on the amount of pneumolysin bound to the membrane and reflects the affinity of the protein for its receptor, cholesterol. A lag occurs before cell lysis begins; this is dependent on oligomerization of pneumolysin. Kinetic dissection of cell lysis by pneumolysin demonstrates the capacity of MACPF/CDCs to generate pore-forming oligomeric structures of variable size with, most likely, different functional roles in biology.

## Introduction

The membrane attack complex-perforin/cholesterol-dependent cytolysin (MACPF/CDC) family is the largest-known group of pore-forming proteins (Anderluh and Gilbert 2014; Gilbert et al. 2013). Members have been identified in every kind of cellular life form apart from the Archaebacteria, and within their producing organisms they enact a plethora of different biological functions (Anderluh et al. 2014). The two family names combined within the current denomination for this group of proteins were initially identified separately. Although their pore structures are superficially similar in ultrastructural appearance (Bhakdi and Tranum-Jensen 1991; Bhakdi et al. 1985; Gilbert et al. 2013; Morgan et al. 1994; Young et al. 1986a), they display no directly detectable sequence homology. The cholesterol-dependent cytolysins (CDCs) of Gram-positive bacteria have long been recognized as pathogenicity determinants of their producing organisms such as *Streptococcus pneumoniae* (producing pneumolysin) (Hirst et al. 2008), *Clostridium perfringens* (perfringolysin) (Awad et al. 2001) and *Listeria monocytogenes* (listeriolysin) (Birmingham et al. 2008; Czuczman et al. 2014). CDCs are adapted to their different producing organisms and to the hosts they, in turn, infect. This was made clear from phylogenetic analysis of their sequences, which displays a clustering mapping onto bacterial genus and bacterial environment (Anderluh et al. 2014). The other branch defining this family of proteins, the ‘perforins’ or ‘MACPFs’ (Gilbert 2015), was first identified in the form of the serum complement membrane attack complex (Borsos et al. 1964; Tschopp 1984) and then in mammalian perforin-1 (Podack and Dennert 1983; Young et al. 1986a), which cytotoxic T lymphocytes and natural killer cells use to deliver a lethal hit to target antigen-presenting cells (Metkar et al. 2015; Voskoboinik et al. 2006). It was, however, only the solution of 3D atomic structures for two MACPF proteins, in 2007, which revealed that MACPFs and CDCs clearly are structurally homologous proteins with a common evolutionary ancestor (Hadders et al. 2007; Rosado et al. 2007). Several structures of MACPF proteins are now known, and these are allowing structurally based phylogenetic studies to be carried out with increasing accuracy (Gilbert et al. 2013, 2014; Gilbert 2014, 2015). However, the separate naming of CDCs and MACPFs must now be seen to be a ‘founder effect’ of the initial identification of two clusters of sequentially similar polypeptides from this very large family of proteins, and not a genuine segmentation into two quite distinct groupings. In reality, there is not only a continuity of structure among the family members (Gilbert et al. 2013, 2014; Gilbert 2014) but also of sequence variation.

**Fig. 1:**
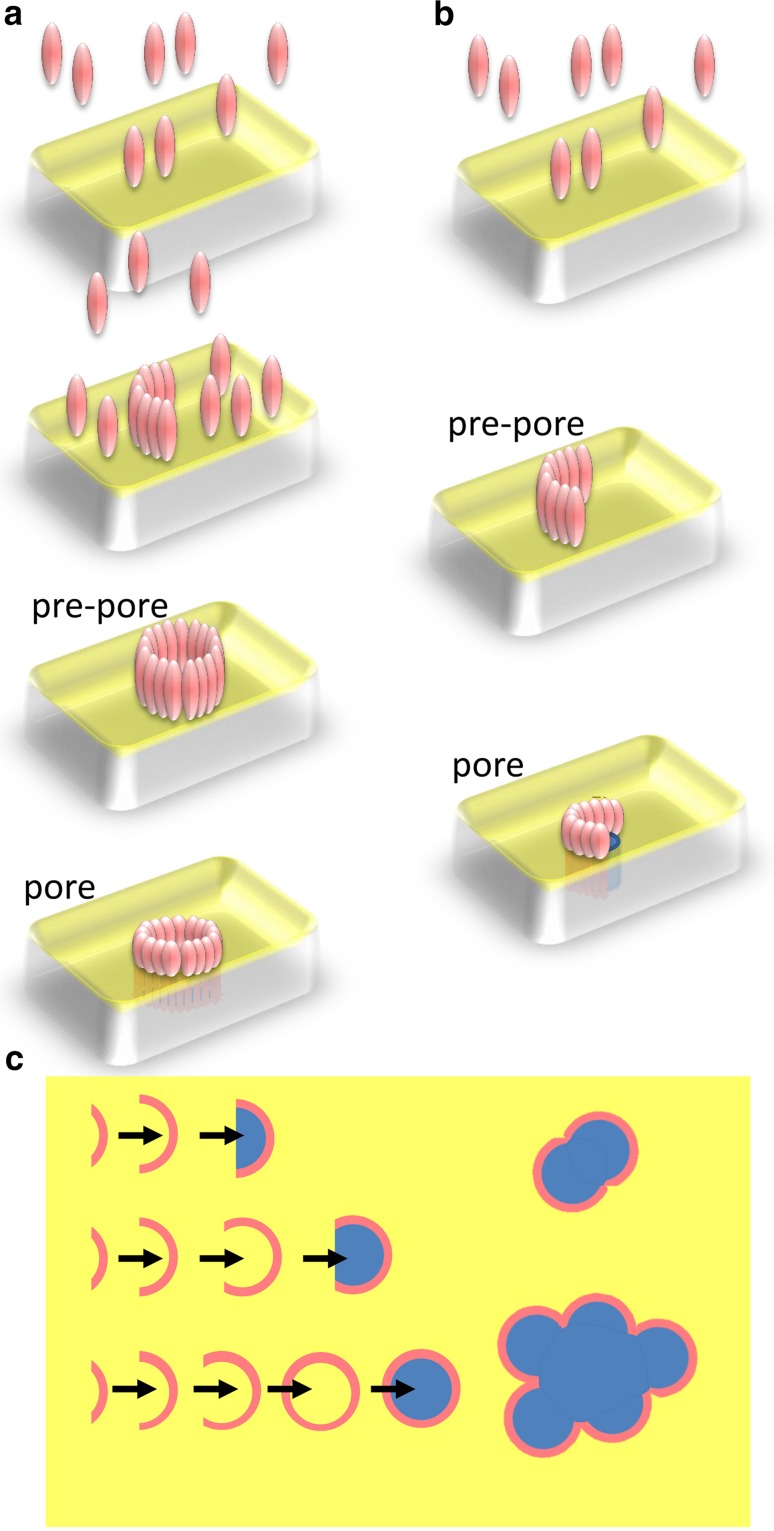
Models of pore formation by MACPF/CDC family proteins. **a** Pore formation by a complete ring of protein subunits—monomers bind to a membrane, assemble on it into a pre-pore, and then undergo a pre-pore to pore transition. **b** Pore formation by an incomplete ring of protein subunits, or arc, as in **a**. **c** Overview of the kinetically governed determination of MACPF/CDC pore size. The size of oligomer formed before the *pre-pore* to *pore* transition caps further assembly and is determined by the concentration of protein available (Gilbert 2002, 2005, 2010; Leung et al. 2014). Once formed, arc pores can associate with each other to form larger lesions (Mulvihill et al. 2015; Podobnik et al. 2015; Praper et al. 2011)

All MACPF/CDC proteins that have been well characterized to date are, broadly speaking, pore-forming proteins. The mechanism of pore formation has been mostly mapped out with CDC family members (Gilbert 2005; Tilley et al. 2005; Tweten 2005), and the available evidence suggests that perforin (Gilbert et al. 2013; Praper et al. 2011) and the perforin-like fungal protein pleurotolysin (Lukoyanova et al. 2015; Ota et al. 2013) share modes of action with them. In this mechanism, monomeric protein binds to a membrane surface and oligomerizes upon it to generate ring-shaped structures, which constitute pre-pore assemblies (Gilbert et al. 1999b; Hotze et al. 2001; Tilley et al. 2005). Pore formation itself ensues when a large conformational change occurs in which a pair of clusters of α-helices refold into a pair of β-sheet hairpins, which then insert into the membrane to form a pore (Czajkowsky et al. 2004; Reboul et al. 2014; Shatursky et al. 1999; Shepard et al. 1998; Tilley et al. 2005) (Fig. 1a). The initial binding to the membrane is, with CDCs, in most cases thought to be based on a direct interaction with cholesterol, although intermedilysin (from the human-specific bacterium *Streptococcus intermedius*) uses CD59 to make initial surface contact (Giddings et al. 2004). Perforin’s contact with membranes depends on calcium activation of its C2-like C-terminal membrane-binding domain but is not thought to be lipid specific. The basis for its failure to also damage the cells that release it is still unknown (Lopez et al. 2013; Metkar et al. 2011, 2015). The complement membrane attack complex is however different from the CDCs and perforin in that its membrane binding depends on the activation and formation of a nucleating C5b-8 complex of proteins. This complex then attracts multiple copies of the principal pore-forming subunit, C9 (Aleshin et al. 2012; Sonnen and Henneke 2014). There may not be a pre-pore oligomeric state for the MAC, in contrast to the CDCs and perforin (Aleshin et al. 2012; Sonnen and Henneke 2014).

In addition to complete rings of subunits forming pores in target membranes, since the earliest days of work on MACPF and CDC proteins there has been a suspicion that incomplete rings, or arcs of subunits, are capable of the same feat (Bhakdi and Tranum-Jensen 1991; Bhakdi et al. 1985; Borsos et al. 1964; Podack and Dennert 1983; Tschopp 1984) (Fig. 1b). This view has continued to be strongly argued (Gilbert 2002, 2005, 2010; Gilbert et al. 2013; Gilbert 2015) but has mostly been neglected (Dunstone and Tweten 2012; Lukoyanova and Saibil 2008) or argued against (Tweten et al. 2015) though the tone of the discussion is changing (Reboul et al. 2016). Among the evidence in favor of the argument that arcs of subunits form functional membrane pores are negative-stain electron microscopy of membranes after MACPF/CDC attack, which have repeatedly shown the presence of protein arcs apparently partly enclosing transmembrane pores with the perimeter completed by the lipid membrane itself (Bhakdi and Tranum-Jensen 1991; Bhakdi et al. 1985; Borsos et al. 1964; Podack and Dennert 1983; Tschopp 1984). In addition, a number of functional studies have also supported pore formation by protein arcs, including the formation of pores with a wide range of conductance values and whose conductance traces are affected by the lipid composition of targeted membranes; the enhancement of lipid “flip-flop” between inner and outer membrane leaflets when pores form; and the imaging of apparent arc-formed pores in situ in tissue sections and on whole cells, which strongly supports their biological relevance (Benz et al. 1986; Felzen et al. 1994; Gilbert 2015; Korchev et al. 1998; Marchioretto et al. 2013; Menestrina et al. 1990; Metkar et al. 2011, 2015; Palmer et al. 1998; Praper et al. 2011; Young et al. 1986a, b, c). Although it might be felt that pores formed at the interface between arcs of protein and membrane bilayer components would be energetically unfeasible, the formation of a partially toroidal structure by the lipids is thought to reduce the line tension and so stabilize the structures, in a way analogous to the formation of purely lipidic toroidal pores during electroporation (Weaver 1994). See elsewhere for further discussion (Gilbert et al. 2013; Gilbert 2015, 2016).

The key contribution of membrane binding to the assembly of MACPF/CDC proteins into pore-forming oligomers is simply the concentration of protein monomers on a plane whereby sufficient interactions are undergone for self-assembly to occur. A variety of data show this, including the demonstration that oligomerization induced in solution is concentration-dependent and can continue indefinitely (leading to helical oligomers rather than the rings found on membranes due to planar constraint) (Gilbert et al. 1998, 1999a, b), that perforin oligomerization can simply be induced by calcium in solution (Metkar et al. 2011), that the size of oligomers can be capped with mutant protein (Palmer et al. 1998), and that disulphide locking of oligomers, which prevents pore formation, affects the distribution of oligomer size (Czajkowsky et al. 2004) (though that was not the inference originally drawn). The observation that oligomer size is affected by the prevention of pore formation has been discussed further elsewhere (Gilbert 2005). This key insight supports a model for the kinetic determination of pore structure whereby the size of oligomeric assembly is determined by the availability of subunits for recruitment into the growing assembly (Gilbert 2002). When an assembly of oligomers terminates with an arc rather than a full ring, it is due to the kinetic trapping of that assembly by a pre-pore to pore transition (Gilbert 2005, 2010). This model has recently been re-proposed and quantified (Leung et al. 2014) and is now considered to be accepted (Mulvihill et al. 2015; Podobnik et al. 2015).

Ultimately, a transition to 3D imaging provided the widely convincing evidence needed that MACPF/CDC proteins form pores using diverse structures—both arcs and rings of subunits. Firstly, cryo-electron tomography and sub-volume averaging allowed the determination of a set of reconstructions of arc and ring assemblies of subunits in pre-pore and pore-forming states (Sonnen et al. 2014). Later, three studies using AFM could image the same assemblies (Leung et al. 2014; Mulvihill et al. 2015; Podobnik et al. 2015), and in the case of the studies on listeriolysin greater ranges of different kinds of assembly to those observed before at this level of detail (Mulvihill et al. 2015; Podobnik et al. 2015) (Fig. 1c). A more recent study of pore formation by streptolysin O using quartz crystal microbalance with dissipation monitoring (QCM-D) has also supported the conclusion that variably sized oligomers (incomplete and complete rings) form membrane pores (Stewart et al. 2015).

In this paper, we describe kinetic data that provide further support for the model of pore formation by rings or kinetically trapped arcs of MACPF/CDC subunits and map out factors determining pore size (Gilbert 2002; Leung et al. 2014; Mulvihill et al. 2015; Podobnik et al. 2015). Although erythrocytes are not thought to be biological targets of MACPF/CDC proteins, saving malaria-associated members of the family (Garg et al. 2013), the red blood cell membrane has long provided a valuable model system for the analysis of pore formation. Bernheimer followed lysis of erythrocytes by a variety of pore-forming agents, observing the pattern now long known for the membrane action of protein toxins: a lag period when lysis did not occur, followed by a rapid lytic event (Bernheimer 1947). In another report, Oberley and Duncan showed how an initial temperature-independent membrane-binding event is followed by lysis, which is prevented at 4 °C because oligomerization and/or pore formation are retarded (Oberley and Duncan 1971). The real-time monitoring of colloid osmotic lysis due to MACPF/CDC pore formation was then established as a way of monitoring the activity of a MACPF/CDC, in particular perfringolysin (Harris et al. 1991). The rapid influx of water into the erythrocyte interior to provide an osmotic balance bursts the red cell membrane and the lysis rate is dependent on the concentration of the pore-forming protein involved and the temperature of the sample. Building on these observations, a variety of kinetic approaches to study MACPF/CDC proteins have been used over the years. Relatively early on, it was shown that the lag period consists of the self-association of subunits to form oligomeric complexes (Harris et al. 1991). The different stages in pore formation were then further defined using mutant forms of perfringolysin in which the pore-forming regions are locked with a disulphide bond so that only pre-pore structures could form (Heuck et al. 2000; Hotze et al. 2001). Binding could be distinguished from oligomerization by their relative timeframes, but both were unaffected by the presence of a disulphide lock and this enabled it to be shown that oligomerization of protein determines the rate of pore formation overall, rather than the pre-pore to pore transition (Heuck et al. 2000; Hotze et al. 2001). The use of liposomes loaded with fluorescent dye-labeled proteins also allowed the real-time monitoring of leakage caused by the activity of perfringolysin, giving a comparative measure of pore formation kinetics (Heuck et al. 2000; Hotze et al. 2002).

Pore formation—or rather, oligomerization—by streptolysin has also been subjected to kinetic studies (Palmer et al. 1995). In a paper in which the sizes of oligomers were quantified using ^125^I-labeled protein, the temperature dependence of oligomerization was made use of to distinguish a first-order binding event [also reflecting the temperature-independent (Oberley and Duncan 1971) 1:1 interaction of CDCs and cholesterol; (Nollmann et al. 2004)] from a second-order process of oligomerization whose kinetic pattern changes with increasing CDC concentration (Palmer et al. 1995). The conclusion made was that arcs of streptolysin subunits are kinetically significant intermediates; this directly relates to the model of pore formation by kinetically trapped arcs of subunits proposed since then (Gilbert 2002, 2005, 2010; Leung et al. 2014). In work using QCM-D, it has also been shown that streptolysin, as a model CDC, binds non-cooperatively to a lipid membrane (Stewart et al. 2015). The use of mutants alongside wild-type protein then demonstrated that arcs of streptolysin were forming and inserting into membranes and were most likely forming pores (Stewart et al. 2015). These data were interpreted with respect to atomic force microscopy images/measurements generated as part of the same study (Stewart et al. 2015) and also using previously published images (Czajkowsky et al. 2004), which were interpreted in exactly the same way as reported earlier (Gilbert 2005). The QCM-D experiments showed that membrane binding, oligomerization, and insertion to form a pore can be kinetically distinguished, and that the lipids within the membrane are, most likely, not lost from it during pore formation (Stewart et al. 2015). This agreed with an insight from cryo-electron tomography and sub-tomogram averaging that lipids flow out from under an inserting CDC oligomer (Gilbert 2016; Sonnen et al. 2014) but contrasts with another study using AFM that suggested lipids are ejected out of membranes on pore formation (Leung et al. 2014).

In the work reported in this paper, a kinetic assay is used to provide data supporting the model of pore formation by MACPF/CDCs previously set out (Gilbert 2002, 2005, 2010) and with which the work of Stewart et al. (2015), as well as that of others (Leung et al. 2014), agrees. Following the dependence of rate of lysis and length of lag before lysis occurs, on concentration of pneumolysin, indicates that the rate is governed by two different kinds of rate-determining processes, whereas the lag length is governed by the same rate-limiting factors at all concentrations of pneumolysin tested. We show that at low toxin concentrations, the rate of lysis is governed by the pre-pore to pore transition of incomplete rings of subunits, while at higher toxin concentrations the rate of lysis is governed simply by the affinity of protein for the membrane. At all concentrations tested, the lag length is governed by the time taken for oligomerization to occur, in agreement with previous findings (Harris et al. 1991).

## Materials and methods

Pneumolysin was expressed and purified exactly as previously described (Gilbert et al. 1999a).

Sheep erythrocytes (in Alsever’s solution; TCS Microbiology Ltd, Botolph Claydon, Buckingham, UK) were centrifuged at 5000 rpm for 1 min in a benchtop centrifuge and the pellet resuspended in 125 mM PBS (8 mM Na_2_HPO_4_, 1.5 mM KH_2_PO_4_, 2.5 mM KCl, 250 mM NaCl, 125 mM NaCl, pH 7.48) to a concentration of 4 % v/v pellet/buffer. Because of imprecision in pipetting a dense solution such as pelleted cells, the 700-nm scatter of the erythrocyte solution was adjusted so that it was always 2.00 ± 0.05. Then, 40 μl pneumolysin at a known concentration, also in 125 mM PBS, was added to a 5-mm pathlength cuvette held in a CE5500 double-beam spectrophotometer (Cecil Instruments, Cambridge, UK) fitted with a real-time printer-plotter, and with the cuvette housing maintained at constant temperature by a circulating heated water bath; 460 μl of 4 % v/v sheep blood at the required temperature were then added to the pneumolysin solution and the resulting cell lysis followed by the decrease in scattered light. Multiple concentrations of pneumolysin were used, at temperatures ranging from 30 to 38.5 °C, and the effect of cell density was also tested with cell suspensions ranging from 1.5 to 2 OD_700_. The experiments were repeated several times, using different pneumolysin preparations, and although there was some variation in the absolute values of rate of lysis, the patterns seen within each set of experiments were constant. The conclusions drawn in this paper relate to the patterns of variation of lysis rate, lag length, and so on, and not to their absolute values. They are thus validated by repeated observation. Data were analyzed using equations described in the Results and Discussion section. All plots were made using the curve-fitting program ProFit (QuantumSoft, Ütikon-am-See, Switzerland).

## Results and discussion

### Primary plots

The kinetic experiments described in this paper constitute a more complete analysis of the concentration dependence of MACPF/CDC pore activity than previously performed, mapping not only the rate of real-time lysis alone (Heuck et al. 2000; Hotze et al. 2002), but also the variation of distinctive features of the real-time lysis curve with protein concentration. It also contrasts with studies that have investigated the MACPF/CDC oligomerization process but not at the same time as the actual functioning of pores (Palmer et al. 1995; Stewart et al. 2015). Our work makes use exclusively of wild-type pneumolysin and relies on the comparison of single-temperature experiments rather than making use of temperature jump approaches. Well-controlled experimental temperatures were ensured by constant maintenance of the reagents at the required value both prior to and after mixing. Our work is therefore distinct from approaches that have used temperature jumps or locking mutants to separate events in cell lysis (Harris et al. 1991; Heuck et al. 2000; Hotze et al. 2001; Oberley and Duncan 1971; Palmer et al. 1995) and complementary to them.

To analyze our data, we sought to apply models for enzymatic activity. However, it is not obvious that cell lysis can appropriately be modeled analogous to enzymatic mechanisms. For example, the standard Michaelis–Menten steady-state model for enzyme activity involves a reversible step in which a metastable transition state, i.e., the enzyme substrate complex, is established, before an irreversible step in which products are formed ensues.

1 $$E + S \Leftrightarrow ES \Rightarrow E + P$$

**Fig. 2:**
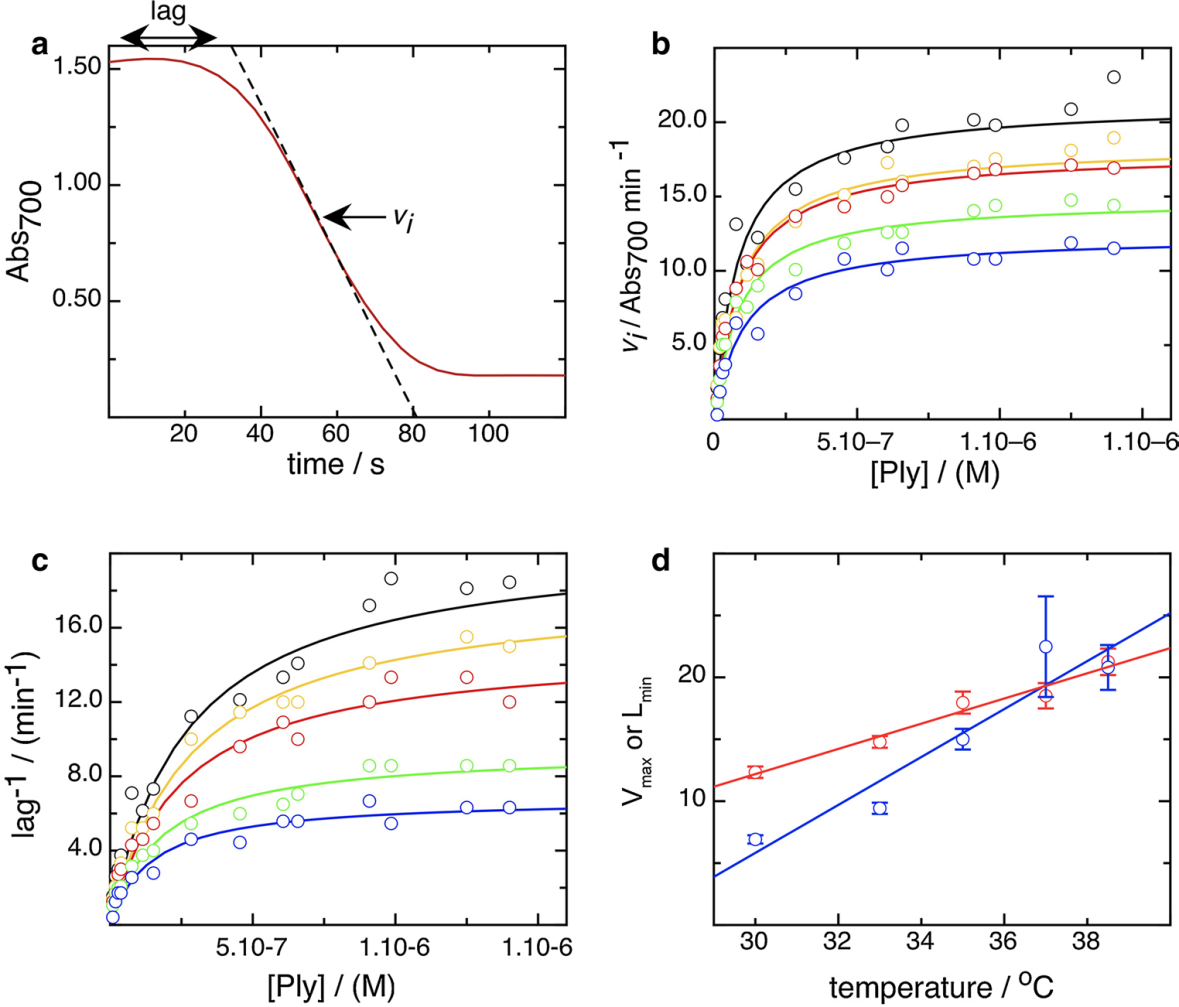
A steady-state kinetic model can be used to model cell lysis. **a** Typical data plot showing constant absorbance (scatter at 700 nm) immediately after mixing toxin and erythrocyte suspension, and then a hyperbolic decline as the cells lyse. The tangent to the point of inflection defines a rate of lysis, *v*_*i*_ and this allows definition of a length of lag, the time from the origin to the tangent, as shown. **b** Plot of rate of lysis, *v*_*i*_, against toxin concentration at 30 °C (*blue symbols*/*line*), 33 °C (*green*), 35 °C (*red*), 37 °C (*orange*), and 38.5 °C (*black*), fitted using Eq. (2), a modification of the Michaelis–Menten equation. **c** Plot of reciprocal lag length against toxin concentration for the same five temperatures as in **b**, with the same *color code*. These distributions have again been fitted with hyperbolic curves, using Eq. (3). **d** Plot of dependence of maximum *v*_*i*_ (*V*_max_) (*blue*) and minimum lag (*red*; expressed reciprocally) on temperature, as computed using the fits shown in **b** and **c**, respectively

It is not obvious that the action of pore-forming proteins involves any reversible steps once membrane binding has occurred (Ohno-Iwashita et al. 1988; Palmer et al. 1995), but the pre-pore could be argued to resemble a transition state. We therefore attempted to study erythrocyte lysis by pneumolysin within a steady-state kinetic model, and found that, in fact, such a model describes the lytic process very well. Using a spectrophotometer to measure the scatter of light by red blood cells, we observed its subsequent decline in the presence of pneumolysin, indicating cell lysis. The decline in scatter was measured in real time (Fig. 2a), and showed two obvious characteristics. Firstly, a lag period during which the cell density remained constant, and secondly a period of rapid cell disappearance. This pattern replicates that seen previously for CDCs (Bernheimer 1947; Harris et al. 1991), but which has not yet been quantified in terms of toxin concentration, temperature, and cell density. We measured a single value for the rate of lysis by taking the inflection point of the fall in cell density, and from this a lag length by taking the time between the origin and the tangent to the inflection point (Fig. 2a). We then plotted the rate of lysis against the concentration of pneumolysin to obtain a curve that could be well fitted by a rectangular hyperbola using a variation of the Michaelis–Menten equation

2 $$v_{i} = \frac{{V_{\text{max} } p}}{{k_{0.5} + p}}$$

where *v*_*i*_ is the rate of lysis, *p* is concentration of pneumolysin, *V*_max_ is the theoretical maximal rate of lysis, and *k*_0.5_ is the concentration at half that rate. We repeated this for a series of temperatures, between 30 and 38.5 °C, as shown in Fig. 2b (output values given in Table 1), and it is apparent that Eq. (2) does model the data well. We also plotted the inverse of the lag length against concentration, on the basis that this would allow us to compare directly rate and lag data, using Eq. (3), a simple variant of Eq. (2)

3 $$\frac{1}{l} = \frac{{\frac{1}{{L_{\text{min} } }}p}}{{k_{0.5} + p}}$$

where *l* is the lag, *L*_min_ the minimal lag length at a particular temperature, and *k*_0.5_ the concentration at twice that value. The lag length data, plotted reciprocally, could be fitted well with Eq. (3) at all temperatures (Fig. 2c), which suggests again that the kinetic model we are using is appropriate for measuring cell lysis in our system (output parameters in Table 1).

**Table 1:** Fit parameters from application of steady-state kinetics to cell lysis

Temperature (°C)	*k*_0.5_ (nM)	*V*_max_ (A_700_ min^−1^)
30.0	102 ± 17	12.3 ± 0.46
33.0	86 ± 12	14.8 ± 0.47
35.0	88 ± 19	18.0 ± 0.89
37.0	91 ± 22	18.5 ± 1.02
38.5	82 ± 19	21.3 ± 1.07

	*k*_0.5_ (nM)	*L*_min_ (minutes)
30.0	143 ± 29	6.9 ± 0.33
33.0	176 ± 31	9.4 ± 0.45
35.0	243 ± 44	15.0 ± 0.83
37.0	405 ± 196	22.5 ± 4.06
38.5	266 ± 7.3	20.8 ± 1.81

The values reported arise from a single set of five experiments performed using the same preparation of pneumolysin on a single day. See “Materials and methods” for discussion of the reproducibility of the assay

### Secondary and tertiary plots

**Fig. 3:**
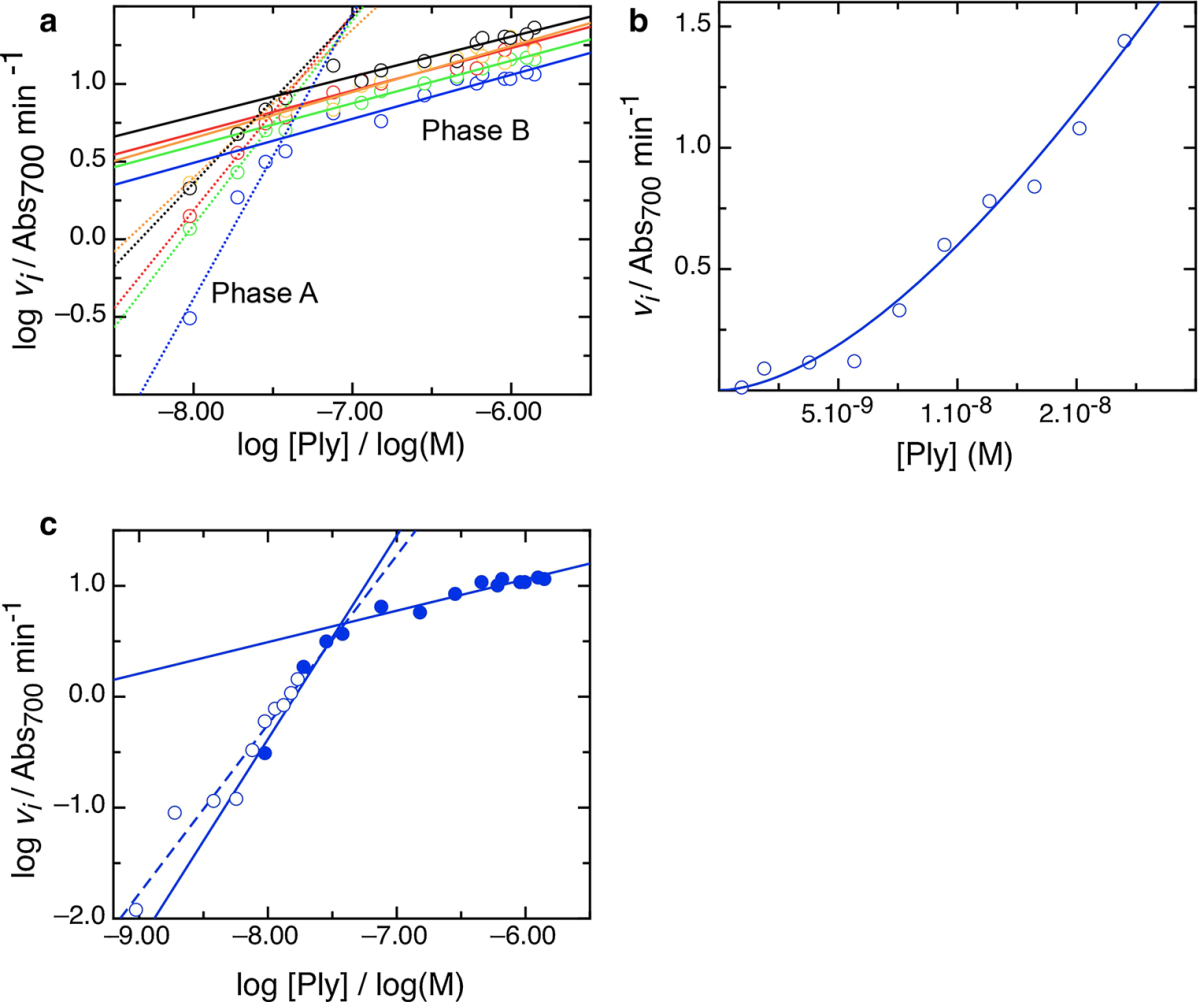
Rate of lysis displays two kinds of concentration dependence, and at lower pneumolysin concentrations is cooperative. **a** Dependence of rate, *v*_*i*_, on concentration at a range of temperatures, displayed by plotting logarithms to calculate the order of the reaction. As in Fig. 2, 30 °C data are colored* blue*, 33 °C *green*, 35 °C *red*, 37 °C* orange*, and 38.5 °C *black*. A *dotted line* fits rate order *phase A*, a *solid line* rate order *phase b*, and their gradients give the actual orders of the reaction. **b** Measurement of *v*_*i*_ over the lower concentration range (rate *phase A*), at 30 °C, to pick out the cooperative kinetics in rate *phase A.* Hill coefficient, *h* = 1.7. **c** Logarithmic plot of the data shown in **b** demonstrating it is within rate *phase A*

Secondary plots of parameters derived from the primary fits shown in Fig. 2b, 2c allow us further to define the mechanistic stages of cell lysis by pneumolysin. In Fig. 2d we show how the *V*_max_ and *L*_min_ vary with temperature, and that they are clearly distinct from each other in the way that a rise in temperature either increases the rate of cell lysis or decreases the lag in time before it occurs. The difference in the kinetic parameters derived strongly indicates that the phases involve different processes governed by separate rate-determining steps, because the temperature-dependent effect differs in degree. Furthermore, plotting the rate and the lag logarithmically, to obtain a measure of reaction order, also shows a difference between them. The rate data (Fig. 3a) show a biphasic order pattern, with a value of 1–2 at lower concentration (“phase A”) and one of ~0.3 at higher concentration (“phase B”) (see Table 2 for order values). An order >1 suggests the possibility of cooperativity which can be simply described by an alternative model to that described by Eq. (2), incorporating an index of toxin concentration, the Hill coefficient, *h*

4 $$v_{i} = \frac{{V_{\text{min} } p^{h} }}{{k_{0.5} + p^{h} }}$$

To ascertain if this applies to rate order A, we measured the kinetics of cell lysis by pneumolysin at a series of lower concentrations and at 30 °C to accentuate any effect. As shown in Fig. 3b, a cooperative trend in lysis is apparent, with a cooperativity coefficient *h* of 1.7. Plotting logarithmically indicates that these data recapitulate the dependence of lysis on concentration already identified as phase A, which is thereby shown to be cooperative (Fig. 3c).

**Table 2:** Dependence of kinetic order on temperature for cell lysis and lag length

Temperature (°C)	Order, rate phase A	Order, rate phase B	Order, lag
30.0	1.42	0.28	0.84
33.0	1.13	0.24	0.41
35.0	1.10	0.24	0.46
37.0	0.80	0.30	0.60
38.5	0.87	0.30	0.47

**Fig. 4:**
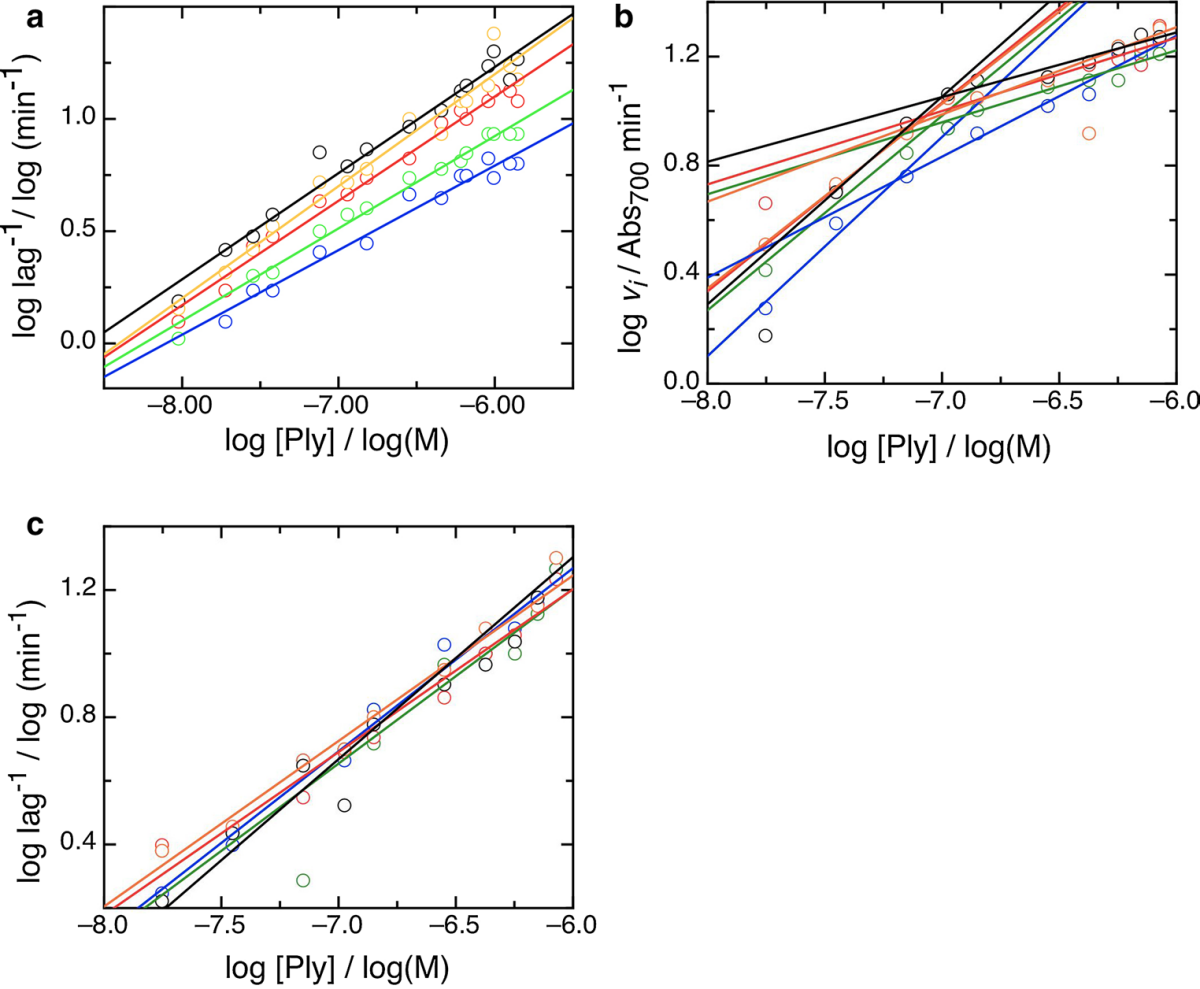
The lag in lysis has constant concentration dependence and the lysis measured is zero order with respect to cell density. **a** Dependence of lag length, expressed reciprocally, on concentration at a range of temperatures, colored as previously with data at 30 °C (*blue symbols*/*line*), 33 °C (*green*), 35 °C (*red*), 37 °C (*orange*), and 38.5 °C (*black*). **b** Dependence of rate, *v*_*i*_, on concentration at a range of temperatures, displayed by plotting logarithms to calculate the order of the reaction. *Blue* at A_700_ = 1.52, *green* at A_700_ = 1.67, *red* at A_700_ = 1.80, *orange* at A_700_ = 1.90 and *black* at A_700_ = 2.00. In each case, rate *phases A* and *B* are fit with *solid lines*; the switch point, *p*_crit_ does not vary systematically with cell density.** c** As **b** for the lag length, expressed reciprocally. The dependence of lag length on concentration of pneumolysin does not alter with cell density in the range tested

In contrast to the rate data, logarithmic plots of the lag data show a single phase (Fig. 4a), with an order of ~0.5. Thus, the lag length is dependent on pneumolysin to the same extent at all concentrations, whereas the dependence of lysis on concentration undergoes a state change at a critical pneumolysin concentration, which we call *p*_crit_, and which does not vary with temperature; for the data shown in Fig. 3a, *p*_crit_ = 60 ± 10.0 nM. A possible rate-determining factor in the rate of cell lysis is the availability of erythrocytes, however as shown in Table 3 and Fig. 4b, *p*_crit_ does not vary significantly with cell density, either (*p*_crit_ = 74.25 ± 9.0 nM), and the lag order maintains a constant value at all cell densities used (Fig. 4c). This means that length of lag and rate of cell lysis is zero order with respect to the availability of membrane surface at the concentrations of pneumolysin measured, and that we can interpret the kinetics we observe in terms of the action of pneumolysin alone, in the manner of a pseudo-first-order reaction.

**Table 3:** Kinetic measurements are zero order with respect to cell density and rate phase B captures cell binding

Cell density, 700 nm	Point of inflection (log M)	Critical concentration, *p*_crit_ (nM)
2.00	−7.15	70.8
1.90	−7.11	77.6
1.80	−7.09	81.3
1.67	−7.09	81.3
1.52	−7.22	60.3

	Temperature (°C)	*K*_d_ (nM)
2.00	30.0	100 ± 20
2.00	33.0	97 ± 15
2.00	35.0	98 ± 27
2.00	37.0	110 ± 30
2.00	38.5	95 ± 27

**Fig. 5:**
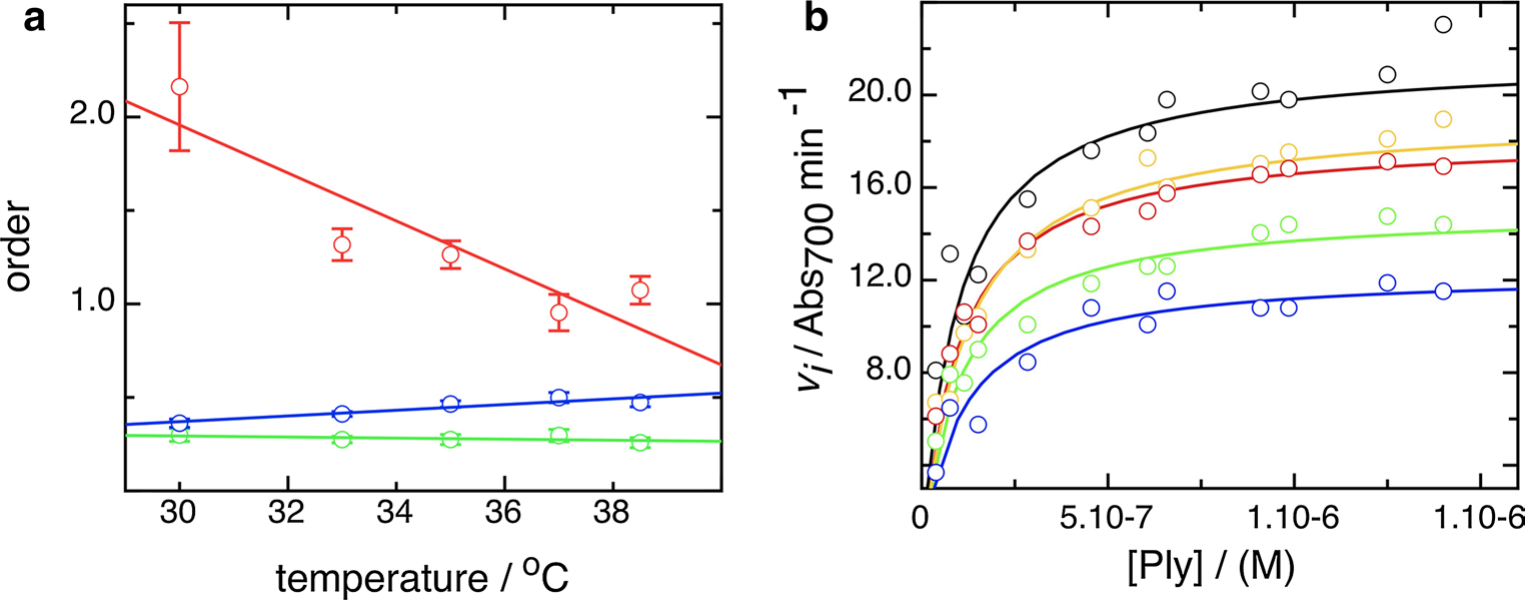
Concentration dependence as a function of temperature and rate *phase B* *K*_d_. **a** Dependence of order on temperature for rate *phase A* (*red*), rate *phase B* (*green*), and lag (*blue*). **b** Plot to determine the *k*_0.5_ for rate *phase B*, and thus the *K*_d_ of pneumolysin binding the membrane. The values computed are listed in Table 3. *Colors* of data and fits are as follows: 30 °C *blue* (*symbols*/*line*), 33 °C *green*, 35 °C *red*, 37 °C *orange*, and 38.5 °C *black*

A plot of the kinetic orders of the two phases in lysis rate and of the lag length with temperature is shown in Fig. 5a. It indicates that the order in rate phase A falls markedly as temperature is raised, while that in rate phase B does not vary significantly with temperature. The order of the lag rises slightly with temperature, which indicates that initiation of lysis by pore formation is actually more concentration dependent at higher temperatures.

We will now seek to identify the molecular processes underlying each of the steps in cell lysis by pneumolysin that we have shown to be distinguishable on the basis of their dependence on toxin concentration and temperature. The known steps in pore formation are (1) binding to the membrane, (2) self-association to form a pre-pore oligomer, (3) pre-pore to pore transition (Gilbert 2005; Hotze et al. 2001; Tilley et al. 2005). Although a pre-pore assembly must form prior to pore formation, it can be an arc of subunits or a complete ring (Gilbert 2002, 2005; Gilbert et al. 2014; Sonnen et al. 2014), with a recent study indicating a minimal arc size of five subunits within the sensitivity of detection (Leung et al. 2014). We will first consider rate phase A, which is cooperative (Fig. 3b, c) and shows a reduction in dependence on pneumolysin concentration with temperature (Fig. 5a). Firstly, binding of CDC molecules to cholesterol is not cooperative (Oberley and Duncan 1971; Stewart et al. 2015) since each protein molecule binds one cholesterol (Nollmann et al. 2004), but reversibly (Ohno-Iwashita et al. 1988; Palmer et al. 1995). Secondly, other studies have suggested that oligomerization itself is not a cooperative process, since the range of oligomeric sizes seen by electron and atomic force microscopy (Bhakdi et al. 1985; Czajkowsky et al. 2004; Leung et al. 2014; Palmer et al. 1998; Podobnik et al. 2015) and that shown by analytical ultracentrifugation (Solovyova et al. 2004) is continuous, and not skewed as would be expected if oligomerization were cooperative. This contention is also supported by the absence of large conformational changes upon oligomerization to a pre-pore (Czajkowsky et al. 2004; Tilley et al. 2005). By exclusion, this suggests that the pre-pore to pore transition is the rate determining process governing rate phase A, and this makes excellent sense in molecular terms since it involves large conformational changes which might be expected to show cooperativity. The cooperative element to the process indicates that the pre-pore to pore transition becomes easier when more toxin is present, i.e., with increasing oligomeric size (increasing arc length). Ring-shaped pneumolysin oligomers have been reported to vary in size (30–40 nm, 38–44 subunits (Tilley et al. 2005)), but the low level of variability observed suggests that oligomerization to complete rings cannot give rise to the cooperativity observed, and we conclude that the pores present at lower toxin concentrations, in particular, include arcs (Fig. 1b, c). It also makes sense in rather simple molecular terms that oligomers will be smaller in size at lower concentrations. Further support for our interpretation is provided by the observation that the dependence of rate phase A on pneumolysin concentration decreases with rising temperature. This indicates that larger pores tend to form at higher temperatures, in line with the kinetic model for pore formation previously developed (Gilbert 2002, 2005, 2010) and now confirmed (Leung et al. 2014) in which the conversion of pre-pore to pore is delayed by the continued accumulation of new subunits to nascent oligomers but can also be undergone by kinetically trapped oligomeric arcs. Thus, more rapid accumulation of subunits allows for larger pores to form, generating larger pre-pores showing greater cooperativity (i.e., less per-molecule dependence) at pre-pore to pore transition. The insight that at higher temperatures larger oligomers form is discussed further below with respect to the lag time before lysis begins.

If the pre-pore to pore transition governs rate phase A, what governs phase B? The order of phase B shows no apparent variation with temperature (Fig. 5a), which suggests that it does not relate to lipid fluidity or the rate of oligomerization on the membrane surface. It has long been understood that MACPF/CDC proteins bind to membranes in a temperature-independent manner (Oberley and Duncan 1971). We suggest therefore that toxin binding to the membrane surface governs rate phase B, and that at higher concentrations of pneumolysin the rate of lysis is dependent on rate of binding (read out as the amount of toxin bound and therefore able to oligomerize and cause lysis). If this is so, then the equilibrium constant (*k*_0.5_ in Eq. 1) of the trend of lysis rate with concentration in this range should give a measure of the *K*_d_ of binding. Figure 5b shows a plot from which values of *K*_d_ were calculated, as listed in Table 3, showing a value of ~10^−7^ M. This is the same as the previously calculated affinity of perfringolysin for cholesterol in membranes (Ohno-Iwashita et al. 1988), further supporting the contention that the rate of lysis in phase B is determined by the affinity of toxin for the membrane.

If rate phase A is governed by pre-pore to pore transition, and phase B by binding to the membrane, what governs the dependence of lag on toxin concentration? We have shown that it must involve distinct molecular processes because of the different orders, dependences on temperature, and pattern of order transitions among the three parameters describing lysis that we have measured. It could be argued a priori that the lag is the time taken for sufficient pores to form to initiate cell lysis, and this would agree with earlier observations that oligomerization is essentially complete before lysis begins (Harris et al. 1991). Due to the existence of a pre-pore stage which can persist for significant periods of time (Gilbert et al. 1999b; Leung et al. 2014; Podobnik et al. 2015; Tilley et al. 2005), *actual* pore formation (i.e. pre-pore to pore transition) is decoupled from the question of how many pores of what size form, in other words from the formation of *sufficient* pores. The formation of sufficient pores is governed by oligomerization, the stage in the three-step mechanism of lysis by CDCs described above but not yet accounted for, and we conclude that the lag period is oligomerization-dependent. In line with this, and the non-cooperative nature of oligomerization to a pre-pore (see above), lag length shows the same dependency on toxin at all concentrations tested. It also shows a slight *increase* in dependency on pneumolysin with temperature, suggesting that larger pores are formed at higher temperatures, in agreement again with the identification of rate phase A as governed by the pre-pore to pore transition and as being *less* dependent on toxin at higher temperatures. Forming a larger pore will take more toxin at higher temperatures but be a necessary consequence of a more rapid migration on the surface of a more energetically dynamic membrane, given a kinetic mechanism for pore formation in which incomplete oligomers (arcs) can get trapped and undergo pre-pore to pore transition (Gilbert 2002, 2005, 2010; Leung et al. 2014; Podobnik et al. 2015). Once formed, however, lysis will be then more rapid because of the increased size of the lesions generated, and the pre-pore to pore transition will itself be less concentration dependent because of its cooperativity (Fig. 3).

In summary, we have shown using pneumolysin as a model system that the dependence of CDC activity on concentration, cell substrate density, and temperature, indicates that all three stages of action (binding, pre-pore oligomerization, and pre-pore to pore transition) are rate limiting at different toxin concentrations, or affect the lag time before lysis rather than the rate of lysis. Taken together, this supports the idea that pore formation by CDC proteins is kinetically governed and that kinetically trapped arcs of subunits form functional pores (Gilbert 2002, 2005, 2010; Leung et al. 2014; Podobnik et al. 2015). Arc pores will form more easily than rings at low toxin concentrations, as supported by our data, and it is likely that the effects of toxins on cells, especially during the early stages of infection, are brought about more by these than by complete rings. Given the similarities between CDCs and MACPF proteins reviewed above, we also hypothesize that our arguments in favor of arciform pores strengthen the case that in perforins, too, unconventional arc-shaped lesion-forming assemblies (Gilbert 2015) as observed by microscopy (Metkar et al. 2015; Young et al. 1986a, 1990) are genuine pores and of physiological significance. This is in strong agreement with single-channel conductance, cell imaging, and cell-labeling experiments (Gilbert 2015; Marchioretto et al. 2013; Metkar et al. 2011, 2015).
